# Use of Silicon Nanowire Sensors for Early Cancer Diagnosis

**DOI:** 10.3390/molecules26123734

**Published:** 2021-06-18

**Authors:** Yuri D. Ivanov, Tatyana S. Romanova, Kristina A. Malsagova, Tatyana O. Pleshakova, Alexander I. Archakov

**Affiliations:** Institute of Biomedical Chemistry, 119121 Moscow, Russia; yurii.ivanov.nata@gmail.com (Y.D.I.); romtatyana@mail.ru (T.S.R.); t.pleshakova1@gmail.com (T.O.P.); alexander.archakov@ibmc.msk.ru (A.I.A.)

**Keywords:** silicon, sensors, biomedicine, nanowire, proteins, microRNA, oncopathology, diagnosis

## Abstract

The review covers some research conducted in the field of medical and biomedical application of devices based on silicon sensor elements (Si-NW-sensors). The use of Si-NW-sensors is one of the key methods used in a whole range of healthcare fields. Their biomedical use is among the most important ones as they offer opportunities for early diagnosis of oncological pathologies, for monitoring the prescribed therapy and for improving the people’s quality of life.

## 1. Introduction

According to the World Health Organization (WHO), the number of deaths from various oncopathology types reached 9.6 mln people in 2018, a number comparable to that of deaths from cardiovascular diseases [1]. During the same year, 272,000 cancer deaths were registered in Russia, whereas 3.7 million people were diagnosed with malignant tumors [2]. We know that oncotherapy at early stages is efficient in 95% of cases [3], however quite often oncological diseases develop asymptomatically, which is why a lot of patients seek help too late.

Molecular diagnostics, as well as histological diagnostics which is the benchmark in oncology, there is a problem of detecting markers associated with the early stage of the disease. Thus, the concentration detection limit, attainable with the use of immunodiagnostic systems, is about 10^−12^ M, which is three orders lower than the concentration of protein markers in blood at an early stage of cancer in case of a tumor sized 1 mm^3^ [4]. 

Existing methods of molecular detection for diagnosing oncological diseases are mainly based on the principles of biospecific detection in the profile of protein molecules and nucleic acids. These methods can be roughly divided into two classes: (1) with labels and (2) without using labels. The labeling method involves implementation of special labels, allowing to amplify signals and attain low concentration detection limits. These include labels based on quantum dots [5], which have a high quantum yield when using probing optical radiation sources, and nanoparticles based on both metallic [6] and nonmetallic structures [7]. Even though labeling methods are highly sensitive [8,9], they are not widely employed due to a number of nonspecific characterististics, such as instability of the nanoparticle aggregation state in biological solutions, the effects of non-specific fluorescence quenching while using fluorescent labels, the problem of labeling efficiency, etc. However, methods without labeling are not characterized by such drawbacks, therefore, they are very promising in the development of new diagnostic systems for detecting oncological diseases at an early stage.

Label-free technologies for earlier detection of diseases can use optical nano-approaches, nanomechanical systems, nanoelectronic systems. Optical systems include systems based on plasmon resonance [8], but these methods are limited by the concentration detection limit of the order of 10^−12^ mol/L. Nanomechanical systems, intended for revelation of diseases, which allow one to attain femtomolar concentration detection limit, can be implemented on the basis of atomic force microscopes [10]; however, these systems have a drawback related to the detection time when the analysis takes several hours. The detection based on nanowire field-effect transistors seems to be the most promising in terms of the concentration detection limit (*DL*) as well as detection time. Nanowire field-effect transistors can be manufactured on the basis of silicon structures as well as other structures, namely, graphene and polyaniline structures. This study focuses on the analysis of silicon nanowire field-effect transistors for the identification of molecular markers associated with cancer, since they are most compatible with standardized technologies for mass production.

The concentration detection limit, attainable using devices relying on silicon sensors that function as field-effect transistors (Si-NW-sensors) is several orders higher (~10^−15^–10^−18^ M) [5,6,7,8,9,10,11], due to the nanometer scale of the sensor. In such sensor platforms, one Si-NW sensor is usually employed in the field-effect transistor as an electric channel between the source and the drain. The size of a nanowire sensitive element is usually from several nm to several microns—namely, a round sensor is several nm in diameter, and the width of nanoribbon sensors can vary from several nm to several microns, while the thickness of the sensor is about 10 nm, which is provided by the standard CMOS technology [3,12,13]. The convenient usage of such sensors is ensured by sensitive elements designed in the form of an array. Devices can contain dozens of sensitive elements [14,15] or whole arrays (about 100 to 200 sensors), which can be modified chemically in order to provide multiplexed, simultaneous detection of several biomarkers [12,14,16,17,18,19,20].

**Figure 1:**
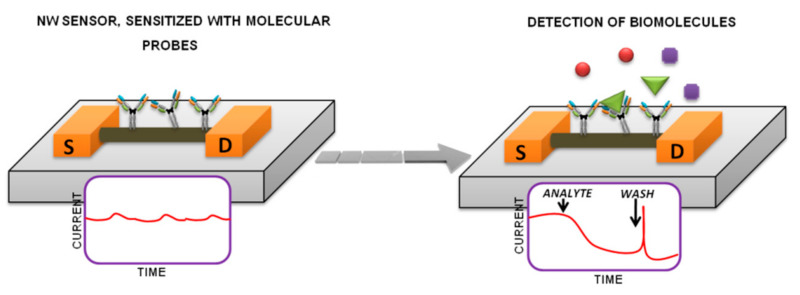
The Si-NW-sensors scheme and representation of the nanowire-based detection of biomolecules. Abbreviations: S–source; D–drain.

The Si-NW-sensors circuit and its working principle are shown in Figure 1.

The operation of such devices is based on the use of the sensor’s surface as a virtual gate: at the adsorption of charged target molecules to the sensor’s surface, the concentration of charge carriers in the subsurface layer of the semiconductor (sensor) changes and its conductivity changes accordingly. To ensure biospecific detection of target biomolecules in an analyte solution, the surface of Si-NW-sensors is normally functionalized by covalent immobilization of molecular probes on their surface. Si-NW-sensors are often used as the bottom of a measuring cell where an analyte solution is placed [16,17,21], or in a microfluidic system [22,23]. In this case, the process of biospecific fishing occurs, and biospecific probe/target biomacromolecule complexes are formed on the surface of sensitive elements. Since the target molecules are charged in a biological fluid, a change in the conductivity of Si-NW-sensors is recorded at a set gate voltage (V_g_), similar to field nanotransistors. Si-NW-sensors can be manufactured in both n- and p-type conduction, and both of these types can be used for the detection of proteins and nucleic acids. Theoretically, upon adsorption on n-type Si-NW-sensors of negatively charged protein or nucleic acid molecules, the conductivity of the sensors decreases and it increases upon adsorption on p-type Si-NW-sensors.

To create Si-NW-sensors based on silicon field-effect transistors, two methods are employed: (1) down-top (vapor-liquid-solid, oxide assisted growth, metal-assisted chemical etching), when coating-catalyzed metals on silicon substrate, coating-catalyzed metals on silicon substrate-laser ablation, Si wafer-coated metal catalyst introduced with Si gas source, oxide-assisted growth-thermal evaporation, oxide-assisted growth-HF, electroless metal deposi-tion-chemical etching are used; and (2) top-down, often with the use of electron-beam lithography and plasma-chemical etching, nanoimprint lithography, DEA technology and photolithography, photolithography-DRIE-TMAH-thermal oxidation, angled thin-film deposition-micrometer scale photolithography, lateral bridging growth. 

Top-down production method is used in CMOS technology (CMOS is a complementary structure of metal-oxide-semiconductor and a standard technology of industrial micro scheme production) [24,25]. Even though there are other methods employed to produce silicon nanowire structures that have a high development potential, e.g., the combination of edge and corner lithography [26], these methods will not be discuss in the paper as the aim of the work is to use these structures for the purposes of biomedicine, not to create them. Even though there are various methods to produce Si-NW-sensors based on field effect transistors, it should be noted that the compatibility between SOI (silicon-on-insulator) nanowire sensors’ fabrication method and CMOS technology is their big advantage as compared to nanowires made of other materials and separately grown nanowires, since they assure a possibility to organize an industrial production of devices, their relatively low cost and small size [13].

The advantage of this method is the possibility to use Si-NW-sensors in medical diagnosis to conduct direct (label-free) highly sensitive multiplex analysis involving a small volume (~5 µL) of biological liquid [27,28]. In biomedical research, nanotubes, graphene sheet or nanowires are often used as sensitive elements in nano biosensors [21,22,23,24,25,29,30].

The use of Si-NW-sensors produced with the CMOS technology appears to be the most [26,27], because it will assure, in the long run: (1) the possibility of a relatively inexpensive mass production; (2) low concentration detection limit (down to a single-molecule level); (3) a possibility of a real-time multiplex analysis (~10 min per bio sample); (4) a high precision of target molecules detection. 

Identifying molecular particularities of the patient’s body with the use of Si-NW-sensors as the disease progresses is the first step towards personalized medicine, whereas their integration in portable devices can revolutionize cancer diagnostics, including a possibility to monitor the patient’s body response to therapy. Thus, the aim of this study was to analyze the application of Si-NW-sensors that are most sensitive to proteins and nucleic acids associated with disease progression.

## 2. Detecting Protein Markers with the Use of Si-NW-Nanosensors

The determination of protein markers associated with oncological diseases given in the literature can be roughly divided into two stages. The first stage is associated with the determination of *DL* detectors based on Si-NW sensors in a model solution in order to determine their potential in the earlier detection of diseases. It should be noted that for earlier detection of diseases, it is essential that the detection limit is below the femtomolar concentration threshold. Almost all the works presented below, with rare exceptions, made it possible to attain such low detection limits using silicon nanowire detectors. This indicates the high potential of this technology for the earlier detection of diseases. The second stage of research involves the adaptation of these technologies to identify protein markers in the patient’s biological fluid, which usually include serum and, to a lesser extent, urine and other biomaterials. More detailed analyses of using Si-NW for the detection of protein markers in pure solutions and in biological material is presented below.

Antibodies or aptamers can be used as sensitive elements by SI-NW sensors for biospecific detection of proteins. First, biospecific Si-NW sensors based on antibodies will be considered.

Lieber C.M. et al. were the first to detect protein and single virus particles with the use of a Si-NW-sensor: a multiplex analysis was conducted of a prostate-specific antigen (PSA), a carcinoembryonic antigen (CEA) and mucin-1, a transmembrane glycoprotein associated with bladder cancer [15,28]. In that study, aldehyde propyltrimethoxysilane (APTMS) was used to modify the surface of the sensor, whereas respective monoclonal antibodies (mAbs) were used to functionalize its surface. The detectable concentration range for PSA, CEA and mucin-1 in buffer solutions was between 50 and 100 fg/mL [15]. (Table 1).

Low concentration detection limits, attainable with Si-NW-sensors, is determined by the increased surface- to-volume ratio [45]. Furthermore, according to literature data, upon decreasing the diameter of the silicon nanowire (Si-NW) from 200 to 50 nm, the concentration detection limit, attainable with the Si-NW-sensor, will become 206 times lower [46]. However, reducing the Si-NW diameter brings about an increase in low-frequency flicker noise that hinders the detection of the valid signal. Another approach, allowing one to lower the concentration detection limit, attainable with a Si-NW-sensor, is the use of electromagnetic fields. In [8], the concentration limit of core antigen of hepatitis C virus (HCVcoreAg) detection, attainable with a Si-NW-sensor, was shifted down to 10^−17^ M by using a microwave generator that allowed to increase the Debye radius up to 14 nm. 

Low limits of CEA detection (1 fg/mL and 10 fg/mL) upon application of a Si-NW-sensor, produced with the standard CMOS technology, were demonstrated in [34]. In another paper [36], low detection limits were attained with a Si-NW-sensor integrated with polydimethylsiloxane microfluidic device (PDMS) [35] when using junctionless nanowire transistor.

Protein α-fucosidase associated with hepatocellular carcinoma [47] was detected at the concentration of ~1.3 pM with the use of the Si-NW-sensor with PDMS microfluid channel, functionalized by the fuconojirimycin receptor (inhibitor of α-fucosidase) [39]. 

Integration of a Si-NW-sensor with two PDMS channels allowed to lower the detection limit for PSA and the marker of the non-small cell lung cancer—19 CYFRA21-1 (cytokeratin fragment) till 1 fg/mL [31].

Cheng et al. used a standard Si-NW-sensor to detect CYFRA21-1 at the concentration of at least 1 fg/mL. The same detection limit was demonstrated by the neuron-specific enolase, an enzyme associated with lung cancer [32]. 

Using the Si-NW-sensor, Pham et al. detected α-fetoprotein associated with liver carcinoma, ovarian cancer and testicular cancer, at the concentration of >10 ng/mL [38].

The protein p16^INK4a^ associated with cervical cancer was detected at the concentration of 100 fg/mL with the use of polycrystalline Si-NW-sensors [44].

For the purpose of bio specific detection of target molecules as molecular probes, not only antibodies but also aptamers may be used [48]. We know that aptamers have a number of advantages compared to antibodies [49]. Their main advantages are temperature resistance, high binding constant, a possibility of a chemical end group modification, simplicity of the synthesis process, a short development period, a possibility to arrange an appropriate in vitro sequence and their low cost [49]. Due to these advantages, aptamers are an attractive object for use in biomedical research, medical diagnostics [48] and therapy [50,51].

In our study, we have shown the possibility of using aptamers as molecular probes to detect D-NFATc1 (nuclear factor of activated T-cells), whose expression in increased in cancer cells, lung tissues, as well as in virus C hepatitis marker HCVcoreAg, at the concentration of 2.5 × 10^−15^ M [7]. Using DNA aptamers we also detected protein–vascular endothelial growth factor associated with breast cancer [52] and gastric cancer [53] at the concentration of 2.59 nM 2.59 nM [40]. 

Using spectral ellipsometry near the observation conditions of surface plasma resonance to experimentally find out the differences between the speed constants of specific interactions of the tumor M2 pyruvate kinase (Tumor M2-PK) in blood serum of the patients suffering from colorectal cancer on different stages and with different metastatic sites and highly specific monoclonal antibodies put in layers on SOI-biochip surface. In this study, upon the detection of Tumor M2-PK, the Si-NW-sensor exhibited low concentration detection limit (10^−15^ M to 10^−13^ M) and high specificity. The obtained results may offer possibilities for creating methods for early colorectal cancer diagnosis, detection of metastases in various areas and identification of disease recurrence [42].

The activated leukocyte cell adhesion molecule (ALCAM) associated with colorectal cancer, pancreatic cancer, melanoma, breast cancer and ovarian cancer [54] was observed in the serum with detection limit of 15.5 pg/mL [43].

It has been shown recently that NFAT is a transcription factor that is highly expressed in tumor cells [5]. Two types of fabricated Si-NW chips: the ones with narrow NW (w = 90 nm) and the ones with wide NW (w = 3 μm) were compared in terms of their multiple use, i.e., a possibility of repeated cycles of detection-regeneration of the Si-NW chips’ surface at the detection of D-NFATc1 in the serum. The analysis has shown that the signal received from the wide NW was a lot more stable than the one obtained from the narrow NW. This confirms that the chips for Si-NW nanosensor containing wide NW are far more suitable for protein analysis in biological liquids. We also showed that the use of Si-NW chips with narrow NW allows one to attain lower detection limits. In this way, the concentration limit of D-NFATc1 protein detection in the serum, attainable using Si-NW chip with wide NWs, amounted to 10^−14^ M, while in the case of Si-NW chip with narrow NWs, the concentration detection limit was much lower (10^−15^ M).

Detection of biomarkers in physiological liquid samples is complicated by such issues as biofouling and non-specific binding, and the resulting need to use purified buffers greatly reduces the clinical relevance of these sensors. In their study, Stern et al. overcame this restriction using distinct components within the sensor to perform purification and detection. The developed approach allows one to isolate Si-NW-sensors from multicomponent medium of the whole blood and reduces its minimum required sensitivity by efficiently pre-concentrating the biomarkers. Researchers have demonstrated specific and quantitative detection of two model cancer antigens, PSA and CA15.3 from 10 µL of the whole blood in less than 20 min [55].

Puppo et al. demonstrated a possibility to use a p-type Si-NW-sensor to detect tumor markers in tumor extracts. This may prove the fact that the created method can be successfully applied to study patients’ biomaterial. In particular, in a multicomponent medium (human breast tumor extract) in the presence of 100000 mass excess of nonspecific protein, exogenously added rabbit antigen in femtomolar range was detected [41].

Apart from the blood serum and blood plasma, urine samples can be used for the analysis. Thus, Chen et al. have carried out a quantitative detection of Apolipoprotein A-II (APOA2 protein) in the urine, the former being a biomarker for bladder cancer diagnosis, with the use of a n-type polycrystalline silicon field-effect transistor. They have shown that such a biosensor efficiently distinguishes mean values of urinary APOA2 protein concentrations between patients with bladder cancer (29–344 ng/mL^−1^) and those with hernia (0.425–9.47 ng/mL^−1^) [37]. Table 1 shows studies that were conducted by different research groups with the use of Si-NW-sensor.

The table shows that at the moment, the range of proteins used to detect oncological diseases is limited. To increase the diagnostics reliability, expanding the range of proteins used on a single chip-array is essential. This approach is promising, since it might contribute to the detection of oncological diseases, as well as to the identification of specific spectra of protein markers responsible not only for the early stage of the disease, but also for metastases, which might begin to form long before the manifestation.

## 3. Detection of Specific Nucleic Scids with the Use of Si-NW-Nanosensors

According to National Institutes of Health (NIH), the main biomarker’s characteristic is the possibility of objective measurement and assessment as an indicator of normal biological processes, pathogenic processes or pharmacological response to therapy [56]. 

The revelation of new biological markers of cancer in order to obtain diagnostic, prognostic or therapeutic data represents one of the key tasks of modern biomedical research. Many tests commonly used in clinical practice, which employ such well-known cancer-associated biomarkers as PSA (prostate cancer marker), CEA (colorectal cancer marker), α-fetoprotein (liver cancer marker), CA 125 (ovarian cancer marker) and CA 19-9 (pancreatic cancer marker), were reported to have insufficient sensitivity and specificity. This appears to be the reason why in July 2012, the US Preventive Services Task Force advised against PSA-based test in prostate cancer screening [57]. Accordingly, new cancer-associated biological markers, which provide better accuracy of the tests, are required to be found in order to reveal tumor progression and changes at the cellular level, and the response of a patient to therapy. Thus, in the recent years, a new group of potential epigenetic tumor markers has been distinguished: microRNA (miRNA) which are non-coding RNAs between 21 and 25 nucleotide-long. The use of miRNAs as diagnostic and prognostic markers has a number of advantages. Most miRNAs are conservative [58]. Furthermore, tumors have unique expression profiles of miRNA already at early carcinogenesis stage [59]. Participation of miRNA in key cell processes including proliferation and cell death, as well as control of oncoprotein expression make them quite promising cancer biomarkers. We know that cancer-specific miRNAs are detected in blood already at early stages of tumor growth, whereas their quantity grows with the progression of disease. In contrast with other biomarker types, miRNA are extremely stable in the blood flow, which makes them most reliable for use in oncology practice [58]. Moreover, miRNAs specific for each cancer type are formed already at early stages of carcinogenesis [59]. 

Contemporary miRNA detection methods are mainly divided into two groups: those based on amplification and hybridization. An example of the former is real-time quantitative PCR (qPCR). The latter includes northern blotting, in situ hybridization, microchip and deep sequencing [60]. In fact, these methods also require amplification of the sample before its hybridization, and do not entirely meet the requirements of clinical diagnosis that are based on the simplicity of application, rapidity of expression, their low cost, high sensitivity and specificity. Today, qPCR is the benchmark of miRNA quantitative assessment [61,62]. However, amplification proceudres are very time-consuming, and qPCR results depend on fluorescent labels. High concentration detection limit and a complex labeling procedures complicate the use of northern blotting and in situ hybridization as routine methods of miRNA detection [63,64]. Restrictions imposed by the use of microarray and deep sequencing are their high cost, long reaction time and sophisticated data anaysis [65,66]. Other methods, such as surface plasmon resonance and Raman spectroscopy, are complicated because of the expensive tools. The use of electrochemical biosensors is limited by their speed of detection and probe knotting effect. In its turn, sensors based on Si-NW have such advantages as label-free detection, low concentration limits of detection, fast response and good selectivity [61].

Upon the anaysis of research papers we have identified a great number of miRNA studies with the use of nanosensors. Thus, in the paper [6] the chip for Si-NW-sensor produced with CMOS-compatible technology using gas-phase recovery and lithography methods, was used to detect oncological diseases in patients based on the analysis of the miRNA rate in blood plasma. It has been shown that Si-NW-sensor allowed to detect an increased miRNA level in patients with mammary gland cancer as compared to the healthy control group and patients with ovarian cancer. 

Lu et al. have developed a complementary metal oxide semiconductor (CMOS)-compatible Si-NW sensor fabricated by an anisotropic wet etching technology with self-limitation which provides a much lower manufacturing cost and an ultra-low concentration limit of detection. This nanosensor has allowed to conduct an express (<1 min) detection of miR-21 и miR-205 with a low limit of detection 1 zmol (~600 copies), as well as an excellent discrimination for single-nucleotide mismatched sequences of tumor-associated miRNAs. Furthermore, with the use of the fabricated Si-NW sensor the researchers managed to detect miRNA in the entire RNA extracted from lung cancer cells, as well as in human blood serum samples, which proves the possibility of their use to identify clinical samples for early cancer diagnosis [67].

In another paper [68] a label-free and direct hybridization analysis for ultrasensitive miRNA detection was created with the use of a Si-NW device. Resistance change measured before and after hybridization correlates directly to concentrations of the hybridized target miRNA. At the same time, the limit of detection of 1fM was shown. This method allows to identify fully identical and non-identical miRNA sequences. Moreover, Si-NW device is capable of detectig miRNA in the entire RNA extracted from Hela cells. Researchers suggest that this approach will allow one to perform label-free detection of miRNA for early diagnosis of cancer with ultra-high sensitivity and good specificity (Table 2). 

The multiplexity of sensor elements on one chip may assure detection of different markers in one bioliquid sample. Gao et al. have presented a CMOS-compatible Si-NW sensor integrated with PDMS microcircuits for label-free and multiplexed real-time miRNA detection with high selectivity, allowing one to attain low concentration limits of detection. It has been shown that this nano sensor identifies in a fast and sensitive way the lung cancer biomarker miRNA-126 with the lowest possible level 0.1 fM and CEA with a good specificity up to 1 fg/mL. The detection ability was defined both in ideal and in clinically significant samples by way of miRNA analysis in the entire RNA and CEA in blood serum [35].

The table below shows the results of studies on the detection of nucleic acids employing a Si-NW sensor. 

The table shows that the studies were mainly devoted to the identification of individual types of microRNAs associated with various types of oncopathologies. To increase the diagnostics reliability, it seems promising to use these technologies to identify the spectrum of microRNAs, rather than individual types, in the analysis of biological materials of patients with oncopathologies, as was noted in the case of protein profiles.

## 4. Conclusions

In this paper, we looked into the importance of the use of Si-NW sensors based on field effect transistors and their application in biomedicine. The use of silicon field transistors to develop sensors offers an advantage to their low signal-to-noise ratio, high thermal stability, resistance to change in response depending on the environment, low concentration detection limits and reliability, high response repetitiveness, minor change in response over time, rapidity of expression and a possibility of their mass production.

Moreover, the integration of Si-NW sensors into microfluidic systems is gaining popularity, since this allows one to use very low (of the order of nanoliters) volumes of samples in the analysis, providing short response times [12,13]. 

Despite the above-mentioned advantages of Si-NW sensors based on field effect transistors, they still have several limitations: (1) well-formed NW structures with controlled atomic composition and heterojunctions are required for the fabrication of devices with good reproducibility; (2) the integration of NW sensors into microfluidic systems can be problematic due to small size of the device; and (3) it is difficult to avoid non-specific interactions upon the use of NW sensors in the analysis of whole blood or serum [74].

Silicon nanowire detectors based on field effect transistors made it possible to achieve low concentration limits of detection of proteins and nucleic acids in sample solutionsat the level of femtomolar and subfemtomolar concentrations. Thus they might be adapted for the purposes of early medical detection of oncological and infectious diseases while overcoming existing problems of nonspecific detection and improving the stability of nanowires’ characteristics in biological environment. It should be noted that the prospects of using such detectors in the earlier detection of oncological diseases will be determined by their adaptation to identify the spectrum of proteins and nucleic acids associated with oncopathologies, rather than individual types of proteins and nucleic acids.

## Figures and Tables

**Table 1:** Detection of proteins associated with various types of oncopathology, using nanowire silicon biosensors.

Analyte	Medium	Detection Limit *	Method	Ref.
PSA	buffer	1.7 × 10^−^^15^ M	Si-NW	[15]
	serum	3.13 × 10^−^^14^ M		
	buffer	3.48 × 10^−^^17^ M	Si-NW PDMS	[31]
	serum	3.48 × 10^−^^16^ M		
	buffer	3.48 × 10^−^^17^ M		[32]
	serum	3.48 × 10^−^^16^ M		
CEA	buffer	6.51 × 10^−^^16^ M	Si-NW	[15]
	serum	1.17 × 10^−^^14^ M		
	serum	6.51 × 10^−^^16^ M	Si-NW PDMS	[33]
	serum	1.3 × 10^−^^16^ M	Si-NW	[34]
	buffer	1.3 × 10^−^^17^ M		[35]
	serum	1.3 × 10^−^^16^ M		
	buffer	1.3 × 10^−^^17^ M	JNT	[36]
MUC1	buffer	4.09 × 10^−^^16^ M	Si-NW	[15]
	serum	7.37 × 10^−^^15^ M		
APOA2	urina	3.8 × 10^−1^^3^ M	Poly-Si-NW	[37]
D-NFATc1	buffer	2.5 × 10^−15^ M	Si-NW	[5]
	serum	2.5 × 10^−1^^4^ M		
CYFRA21-1	buffer	3.33 × 10^−17^ M	Si-NW PDMS	[31]
	serum	3.33 × 10^−1^^6^ M		
	buffer	3.33 × 10^−17^ M	Si-NW	[32]
	serum	3.33×10^−1^^6^ M		
AFP	buffer	1.46 × 10^−1^^3^ M	Si-NW	[38]
	serum	7.28 × 10^−15^ M	Si-NW PDMS	[33]
α-fucosidase	buffer	1.3 × 10^−12^ M	Si-NW PDMS	[39]
VEGF	buffer	2.59 × 10^−^^9^ M	Si-NW	[40]
	tissue	5.0 × 10^−15^ M		[41]
Tumor M2-PK	buffer	10^−13^–10^−15^ M	Si-NW	[42]
ALCAM	serum	2.38 × 10^−1^^3^ M	Si-NW	[43]
p16 ^INK4a^	buffer	6.48 × 10^−1^^5^ M	Poly-Si-NW	[44]

* For comparison, the detection limit presented in the studies has been converted into molar concentrations (M). Abbreviations: Si-NW–silicon nanowire; JNT–junctionless nanowire transistor; PDMS–polydimethylsiloxane; ALCAM–activated leukocyte cell adhesion molecule; AFP–alfa fetoprotein; CEA–carcinoembryonic antigen; CYFRA21–type I cytoskeletal 19; D-NFATc1—nuclear factor of activated T cells 1; MUC1—mucin 1; PSA–prostate-specific antigen; p16 ^INK4a^—cyclin-dependent kinase inhibitor 2A; Tumor M2-PK–pyruvate kinase type M2; VEGF–vascular endothelial growth factor.

**Table 2:** Detection of nucleic acids, using nanowire silicon biosensors.

Analyte	Medium	Detection Limit *	Method	Ref.
DNA	buffer	1.0 × 10^−^^15^ M	Si-NW-FET	[69]
	buffer	1.0 × 10^−^^1^^5^ M		[70]
	buffer	1.0 × 10^−^^1^^5^ M		
miRNA	buffer	1.0 × 10^−^^17^ M	Si-NW	[71]
U6 snRNA	cells	~2.2 × 10^−5^ M *	poly-Si-NW FET	[72]
miRNA	buffer	1.0 × 10^−^^1^^5^ M		
miRNA-363	buffer	1.0 × 10^−^^1^^7^ M	Si-NW	[73]
	plasma	no data		

* To calculate the molar concentration, the molecular weight of U6 snRNA was obtained from the resource https://www.uniprot.org/, (accessed on 11 March 2021).

## Data Availability

Not applicable.
